# Dignity and the Importance of Acknowledgement of Personhood for People With Disability

**DOI:** 10.1177/10497323231204562

**Published:** 2023-10-30

**Authors:** Kelsey Chapman, Angel Dixon, Carolyn Ehrlich, Elizabeth Kendall

**Affiliations:** 1 The Hopkins Centre: Research for Rehabilitation and Resilience, 5723 Griffith University, Nathan, QLD, Australia; 2 Inclusive Futures: Reimagining Disability, 5723 Griffith University, Southport, QLD, Australia

**Keywords:** dignity, disability, acknowledgement, personhood, accessibility, inclusion, human rights

## Abstract

Exploring the intricate relationship between individual and collective experiences, this study explores dignity from the perspectives of people with disability. Using an extreme citizen science approach, we engaged people with disability as active partners in gathering data through qualitative surveys and focus groups. Framework Analysis was employed to ensure the validity of findings while privileging the voices of people with lived experience of disability. Dignity was contingent on the acknowledgement of personhood and the delivery of human rights. Our research identified five key aspects to maintain and protect dignity: (1) acknowledging personhood; (2) recognising people with disability as decision-makers of their lives; (3) realising the right to access information; (4) maintaining the right to privacy; and (5) eliminating or minimising barriers to accessibility and inclusion. Undignified experiences that resulted from a lack of acknowledgement negatively affected participants’ wellbeing, and healthcare settings were identified as particularly challenging environments for dignity. These findings have significant implications for healthcare systems and services within an international and interdisciplinary context. They emphasise the need for adaptable, flexible services, co-designed with people with lived experience of disability. Addressing organisational constraints, resource limitations, and expectations is paramount to ensuring dignity is maintained through the acknowledgement of personhood and safeguarding of human rights.

## Introduction

Disability is a contestable concept with a long history characterised by marginalisation, exclusion, and discrimination. Historically, the medical model of disability has portrayed people with disability as burdens with a flaw or failing of the body that needed to be eradicated or cured (Arstein-Kerslake et al., 2020; Berger & Lorenz, 2016; Hosking, 2008; Levine & Karner, 2023; Rioux & Valentine, 2006). The individual was responsible for their disability. Following the rise of the disability rights movement in the 1970s and '80s, the social model of disability redefined disability as a result of a mismatch between impairment(s) and either physical or social environments, norms, and structures (Arstein-Kerslake et al., 2020; Lawson & Beckett, 2021; Rioux & Valentine, 2006; Vehmas & Watson, 2020). This shift was critical to the advancement of opportunities for people with disability. However, contemporary disability activists and scholars are now moving beyond the social model, to a human rights model of disability. This model extends on the social model to acknowledge the material realities of impairment as a source of pain, fatigue, or functional loss that may lead to disadvantage (Barclay, 2019; Goodley, 2017). It demands that every person with disability has the right to participate in society, to be respected, and to benefit in the same way as people without disability.

We ascribe to the definition of disability articulated in the United Nations Convention on the Rights of Persons with Disabilities (2006). The CRPD states that disability is an ‘evolving concept’ that includes those who have long-term physical, mental, intellectual, or sensory impairments which in interaction with various barriers may ‘hinder their full and effective participation in society on an equal basis with others’ (United Nations, 2006, p. 2). The CRPD also establishes the importance of shifting normative views of people with disability from objects of medical treatment, charity, and pity towards fully fledged members of the society with equal dignity and human rights to those without disability (Shekhar & Fahmy, 2015). The CRPD can provide a guide for rights-based approaches, while still allowing for the consideration of the ‘bodily dimensions of both disablement and impairment,’ as well as intersectional experiences (Shakespeare & Watson, 2022). Importantly, a human rights model of disability establishes the critical importance of dignity for all people.

Dignity, like disability, is a contestable concept. Dignity defines the worth and value of people (Davis, 2021) and has been historically used in language and discourse for more than 2500 years (Shekhar & Fahmy, 2015). Despite its use throughout history and in present day, dignity is a complex, multi-faceted concept with a variety of legal, bioethical, philosophical, and practical applications (Lind et al., 2014). There are two dominant constructs of dignity: inherent and contingent (Jacobson, 2009; Killmister, 2017; Lohne et al., 2010). These constructs are considered independently, as dignity cannot be both inherent – intrinsic and unchangeable – and contingent – dependent, changeable, and not guaranteed. However, an amalgamated view that acknowledges the interplay between the two constructs is emerging (Killmister, 2020).

Inherent dignity is a universally applicable construct that is inalienable and belongs to every human by virtue of their humanness (Allan & Davidson, 2013; Gilabert, 2018; Jacobson, 2009; Jones, 2015; Killmister, 2020; Lohne et al., 2010; Nordenfelt, 2004; Van der Rijt, 2017). Inherent dignity cannot be violated, frustrated, lost, nor earned through rationality, autonomy, or agential action (Allan & Davidson, 2013; Killmister, 2020; Van der Rijt, 2017). However, critics of inherent dignity contest that ‘dignity is not a forgone conclusion for anyone’ and cannot be applied universally (Killmister, 2020, p. 17). Scholars widely accept that dignity can be violated, both through extreme acts like torture and humiliation, as well as through smaller, though no less impactful types of transgressions (Gilabert, 2018; Killmister, 2020). If dignity is not inherent, then it stands to reason that it must be contingent.

Contingent dignity is considered a construct experienced through interactions and influenced by personal, cultural, societal, and relational factors (Jacobson, 2009; Killmister, 2020; Lohne et al., 2010). Subjective factors influence interactions with other people, systems, and structures which dictate required behaviour, respect, and standards, through which dignity can be earned or lost (Killmister, 2020). Contingent interpretations of dignity are related to power constructs like integrity, identity, and status within the society (Gilabert, 2018; Jones, 2015; Nordenfelt, 2004). Scholars widely recognise that dignity is violated and even lost when personal values and preferences are not or cannot be upheld in interaction with other individual, societal, and cultural structures (Clark, 2010; Nordenfelt, 2004; Rejno et al., 2020). Interactions and experiences that feature power imbalances, where an actor is in a position of vulnerability, increase the likelihood of a violation or loss of dignity (Clark, 2010; Jacobson, 2009; Lohne et al., 2010). There is little research that explores how dignity can be regained after it is violated or lost. Once lost, the lack of dignity can leave people vulnerable to ongoing poor treatment and marginalisation (Van der Rijt, 2017), which is the current reality for many people with disability.

The contestable nature of dignity, the tension between dignity being either inherent or contingent, but not both, and the lack of clarity about dignity, has led some to label it as a meaningless, irrelevant construct, even in the context of human rights discourse (Kirchhoffer, 2011; Macklin, 2003). However, dignity is embraced by most national and international human rights frameworks (Chapman et al., 2022a). Researchers have attempted to resolve these criticisms by considering the meaning of dignity as it applies to a particular practice such as palliative care (Chochinov 2002, 2007), dementia services (Kracmarova et al., 2022), and healthcare (Frounfelker & Bartone, 2021; Lind et al., 2014; Philipp et al., 2016; Podolinska & Cap, 2021). Although these examinations include lists of the barriers and enablers to dignity in individual contexts, little is known about how dignity is understood, defined, and experienced by marginalised populations, particularly people with disability (Lohne et al., 2010). This research aimed to address gaps and criticisms in the contemporaneous literature, using extreme citizen science in partnership with people with disability, to uncover how dignity is understood, defined, and experienced by people with disability in their daily lives and as they interact with essential systems.

## Methods

When considering how to approach research exploring the concept of dignity, we could not, in good conscience, do so without upholding dignity throughout the research process. Despite this seemingly obvious statement, few researchers use inclusive participatory methods in partnership with people with disability. The research team included researchers without disability, a researcher with long-term experience of disability personally and as a carer (EK), and a citizen scientist with lived experience of disability (AD) who has research and advocacy experience, to direct and lead all aspects of the research. However, prior to commencing, the entire research team spent time working with people with disability to explore and develop a framework for dignified research (as described in Chapman et al., 2022b).

The Dignity Project Framework for extreme citizen science established four principles of importance to guide inclusive research: (1) using a human rights concept of disability; (2) eliminate barriers to participation; (3) diversity in engagement through accessibility and inclusion; and (4) working transparently (Chapman et al., 2022b). These principles informed and guided this study, including the use of the definition of disability as described in the introduction; the use of user tested and diverse modes for participant participation, as described below under the subheading ‘data collection’; and the embedding of a citizen scientist with disability in the research team (AD).

The Dignity Project Framework includes five phases (vision, uncover, discussion, critical reflection, and change) (Chapman et al., 2022b). Each phase includes suggested tasks to be completed collaboratively with professional researchers and citizen scientists. During the ‘vision’ phase, the research team clearly negotiated roles through governance meetings; developed and published values and commitment documents publicly online; and established flexible work patterns, including work from home and virtual meetings. The final phase ‘change is ongoing, including dissemination of findings. The remainder of the phases of the framework are discussed throughout the Methods section. Using inclusive research methods, namely extreme citizen science, enabled the research team to privilege the perspectives of people with disability throughout all aspects of the research. Inclusive research methods were not previously mentioned by most researchers examining dignity and disability and differ from standard approaches to research. Ethical approval was received from Metro South Human Research Ethics Committee (HREC/2019/QMS/58929) and Griffith University Human Research Ethics Committee (2020/041). The ethical principles of the Declaration of Helsinki were followed, and informed consent was obtained from all participants.

### Participants

Eligible participants included any adult, living in Australia, who self-identified as a person with disability or impairment. Informed consent was obtained in written form for both the survey and focus groups. Family members of people with disability who did not identify as a person with disability themselves were unable to participate. Participants who were not typically verbal could ask for supported participation, although no one expressed interest in this option.

People with disability were purposively recruited via email databases through the partner organisation Queenslanders with Disability Network and through the Health Consumers Queensland. A total of 17 participants participated in an initial short survey. After the survey had been closed, the de-identified data was discussed in detail with another five citizen scientists through a focus group (three of these participants had previously completed the survey). All participants and citizen scientists’ characteristics are provided in Table 1.

**Table 1.** Participant Sample Characteristics.

Participant characteristics	Online survey, *n* (%)	Focus group, *n* (%)
Gender		
Male	7 (41)	2 (40)
Female	10 (59)	3 (60)
Age		
18–29 years	1 (6)	1 (20)
30–39 years	2 (12)	1 (20)
40–49 years	5 (29)	1 (20)
50–59 years	3 (18)	2 (40)
60–69 years	4 (24)	
70–79 years	2 (12)	
Education		
Secondary school only completed	2 (12)	0 (0)
Some university courses completed	2 (12)	0 (0)
Trade/technical/vocational	3 (18)	1 (20)
Bachelor’s degree	6 (35)	2 (40)
Master’s degree	4 (24)	2 (40)
Current employment		
Employed full time	4 (24)	0 (0)
Employed part time	1 (6)	1 (20)
Employed casually	1 (6)	1 (20)
Not currently employed	6 (35)	1 (20)
Self-described	5 (29)	2 (40)
Impairment or disability (select all that apply)		
Physical	9 (53)	4 (80)
Cognitive	3 (18)	1 (20)
Sensory	2 (12)	0 (0)
Hidden	1 (6)	2 (40)
Chronic pain/illness	1 (6)	3 (60)
Mental health		2 (40)
Multiple	1 (6)	
Living arrangements		
Living independently	6 (35)	3 (60)
Living with family	3 (18)	1 (20)
Living with partner	8 (47)	2 (40)
Support arrangements		
Formal support	9 (53)	1 (20)
Informal support	1 (6)	2 (40)
No support	7 (41)	
Combination of informal and formal support	0 (0)	2 (40)

### Data Collection: ‘Uncover’ Phase

Data collection occurred over four time points (an initial short survey conducted at two time points – July and November 2020 – and two small focus groups where data were analysed in more detail). Qualitative interviews were originally proposed, but a COVID-19 lockdown necessitated the use of an online survey. The survey included 21 questions that covered demographic information and questions about definitions of dignity, the experience of dignity, and violations or threats to dignity in all aspects of their lives. Following the demographic questions, the remaining survey questions (see Table 2) were open-ended text responses, where participants could type and upload a word document, audio file, or video recording in response to the question, aligning with the principles of the Dignity Project Framework, which calls for diverse ways of participating to capture the breadth and depth of disability experience (Chapman et al., 2022b).

**Table 2.** Non-Demographic Survey Questions.

Question	Description	Response type
13	When you think of the word dignity, what things come to mind? What does it mean to you?	Text box, with option to upload file (.doc, .pdf, .mp3, or .mov)
14	Please describe any personal experiences, interactions, or moments in your life where you experienced a lack of dignity or something that was undignified?	Text box, with option to upload file (.doc, .pdf, .mp3, or .mov)
14.1	Reflecting on those experiences, what do you think led to it or caused it to happen (including attitudes, laws, and accessibility)?	Text box, with option to upload file (.doc, .pdf, .mp3, or .mov)
14.2	How did these experiences, interactions, or moments of indignity make you feel?	Text box, with option to upload file (.doc, .pdf, .mp3, or .mov)
15	Please describe any personal experience, interactions, or moments in your life where you experienced dignity or something that was dignified?	Text box, with option to upload file (.doc, .pdf, .mp3, or .mov)
15.1	Reflecting on those experiences, what do you think led to them or made them happen (including attitudes, laws, and accessibility)?	Text box, with option to upload file (.doc, .pdf, .mp3, or .mov)
15.2	How did these experiences, interactions, or moments of dignity make you feel?	Text box, with option to upload file (.doc, .pdf, .mp3, or .mov)
16	Thinking more generally, what aspects or elements of an experience, interaction, or moment make you feel as though your dignity is most respected?	Text box, with option to upload file (.doc, .pdf, .mp3, or .mov)
17	What do you think are the most important changes that could be made or things that could be done to increase and improve the quality and frequency of dignified experiences for people with disability?	Text box, with option to upload file (.doc, .pdf, .mp3, or .mov)

The research questions and the online survey platform were pilot tested by five citizen scientists, some of whom were participants in the research and reviewed by the Centre for Accessibility. Participants were able to choose the method of responding that best suited them, to increase accessibility and comfort (e.g. they could upload supporting documentation, provide non-explicit multi-media, or type directly into the text boxes provided as per Chapman et al., 2022b).

Following the completion of the data analysis from the survey (as described below), two semi-structured online focus groups were conducted in May 2021 via Microsoft Teams to ensure safe and flexible mechanisms for participation during COVID-19 lockdown (Chapman et al., 2022b). The research team decided to hold focus groups as the survey data illustrated mostly individual circumstances, emotions, and perspectives, with some insight into broader, shared community experiences (Lambert & Loiselle, 2008). The aims of the focus groups were to identify shared experiences, confirm survey findings for completeness (Lambert & Loiselle, 2008), and specifically prompt questions that examined the theme of acknowledgement (see Table 3). Citizen scientists in the focus groups were encouraged to reflect on the types of interactions and environments where they did and did not experience dignity. Strategies and practices for promoting dignity for people with disability were also discussed.

**Table 3.** Focus Group Prompt Questions.

Question	Description	Relevant theme/finding from survey
1	How do you know when you’re being treated as a human (acknowledgement) and being treated with dignity?	Acknowledgement of personhood – needed some more specific examples of what that means and looks like.
2	What does the experience of dignity and being recognised as a human (acknowledgement) rely on?	Acknowledgement of personhood – policy, structural examination.
3	What is the impact of dignified and undignified treatment of people with disability?	Impact (positive and negative).
4	What sorts of practices would contribute to the promotion of dignity for people with disability?	Solutions and practical forms of acknowledgement.

### Data Analysis: ‘Discuss’ Phase

Coding and data analysis were conducted collaboratively led by KC and AD to ensure the perspectives of lived experience were privileged in the development of findings (Chapman et al., 2022b). Framework Analysis (Ritchie & Spencer, 2002) was used because it supports collaborative and inclusive analysis practices through both inductive and deductive analysis. Framework Analysis follows a five-step process that involves a thorough and context-specific systematic analysis: (1) familiarisation; (2) developing a thematic framework; (3) indexing; (4) charting into headings and subheadings; and (5) mapping and interpretation (Ritchie & Spencer, 2002).

All researchers first familiarised themselves with the qualitative survey data by reading through responses to identify recurring themes. AD and KC then created and refined provisional codes based on both indicative themes, a priori themes and topics, and participant experiences (Ritchie & Spencer, 2002). All survey responses were then indexed using the NVivo^TM^ software package (QSR, 2020). Specific attention was paid to the ways in which participants described dignity in their interactions with systems and services. The data and themes were discussed with the full research team at regular intervals. Collaborative charting was led by AD and KC, in which themes were organised into headings and subheadings with reference to participant responses to build a final thematic framework.

Following the completion of the survey data analysis, the thematic framework was used to guide the development of focus group questions. The focus groups were transcribed and analysed using the same process of Framework Analysis, as described above (Ritchie & Spencer, 2002). However, a separate coding framework was generated and used.

In the last step, the research team used Miro (an online whiteboard tool) to bring together the thematic frameworks from both the survey and focus groups to map and discuss themes, associations, and patterns, which led to the final range of results (Ritchie & Spencer, 2002). The mapping made collective sense of individual experiences, within the context of societal norms and values (Lambert & Loiselle, 2008; Ruiz, 2017).

### Critical Reflection Phase

Reflection, as outlined in the Dignity Project Framework, is an important part of critically appraising research and positionality of the research team (Chapman et al., 2022b). The non-disabled researcher leading the data analysis maintained a digital reflection journal throughout the analysis and publication drafting, as a form of reflexivity (Braun & Clarke, 2012; Fischer, 2009). Daily prompts were used in the journal, including outlining aims, barriers or pain points, use of language, and questions. It was also important for the citizen scientist with disability to maintain reflexivity, particularly as results conflicted or affirmed her lived experience. For example, some participants used language that may be confronting or even offensive to the citizen scientist, which was debriefed and discussed collaboratively with KC and the rest of the team as required, in order to separate personal experience from what was actually contained in the data.

## Results

Dignity is highly subjective and impacted by a diverse range of individual, systemic, and societal factors. Examining both individual and collective experiences resulted in a critical overarching theme of acknowledgement of personhood and a series of related themes, including acknowledgement of modifying personal factors and indignity and lack of acknowledgement. Additional themes were closely aligned with human rights, specifically articles within the CRPD, as strategies for maintaining and enhancing dignity through acknowledgement of personhood, including (1) acknowledgement as decision-maker (CRPD Article 12); (2) right to access information (CRPD Article 21); and (3) right to accessibility and inclusion (CRPD Articles 9 and 19) (United Nations, 2006). These themes are presented in the remainder of the Results section.

### Acknowledgement of Modifying Personal Factors

Individual aspects such as personality, socio-economic status, education, background, and length of time living with disability all impacted in diverse ways on participants’ perspectives on dignity. For example, two participants discussed the importance of religion in relation to their views of dignity as being inherent: ‘Christianity made a fundamental difference for people like me, because of its teachings about looking after the poor and broken’ (Participant 2). Some participants expressed a great deal of anger and frustration about violations of dignity, whereas others were more resigned about the barriers they encountered: ‘A situation that some might see as being undignified and take offence to others will see just a part of living life with disability and brush it off’ (Participant 16). Some participants spoke of employment as an important mechanism for enhancing dignity: ‘My colleagues have supported me and treated me as an equal and valued member of the team’ (Participant 17). Others felt that employment was an entirely undignified experience, with encounters of bullying and harmful attitudinal barriers: ‘When I could finally retire it was like heaven. I had become deeply afraid of my boss doing me wrong’ (Participant 2). Social isolation was described as an undignified outcome for some participants: ‘Makes me feel “less than” and more inclined to stay home’ (Participant 15), whereas others used social boundaries and isolation as a way to maintain their own dignity and decrease the likelihood of undignified interpersonal interactions: ‘I find short brief uncomplicated encounters leave me with more dignity’ (Participant 13). Although the primary aim of this research was to examine how dignity manifested in interactions with systems and services, personal factors were important in defining a person’s perception of dignity.

Identity was a modifying personal factor which impacted dignity, especially for participants who were transitioning to a new identity following the onset of an impairment. Some participants who had recently acquired their impairment felt they needed time to come to terms with their new reality. One participant commented that prior to acquiring an impairment, they ‘didn’t really think about it [dignity] at all’ and ‘took it for granted’ (Participant 3) that they would be treated with dignity. The change in personal identity following an impairment increased some participants’ feelings of vulnerability, but also changed how they viewed dignity – from something that was expected and typically given (inherent) to something more tenuous (contingent). Change in identity highlighted the ways in which dignity can be both inherent and contingent, depending on the personal factors of an individual.

### Acknowledgement of Personhood Is Critical for Dignity

Participants were asked to describe what dignity meant to them and how they experienced it. One participant commented that ‘dignity is central to personhood’ (Participant 8). Many participants equated dignity with the feelings they felt after being treated with dignity, like respect, safety, and independence. Fifteen participants commented that they experienced dignity when they were acknowledged as a human being with worth and value: ‘genuine acknowledgement of existence, who I am’ (Participant 13), ‘treating my life as having worth’ (Participant 3), and being ‘seen and validated as a “real” person’ (Participant 4). For 11 participants, dignity also meant acknowledgement of their diversity, the realities of their life with impairment, and the fluctuating nature of impairments.

Dignity was described by participants as a mix of both the inherent and contingent types. However, most participants focused primarily on the importance of the inherent nature of personhood, something that is often denied to people with disability. Although personhood was viewed as an inherent quality, participants commented on how its dependence on acknowledgement meant dignity was largely contingent in reality. Thus, participants articulated a hybrid conceptualisation of dignity, whereby dignity is contingent on the acknowledgement of the inherent nature of someone’s personhood.

Although dignity was primarily contingent on acknowledgement of personhood, what was acknowledged about a person and how it was acknowledged were also critical. Some participants wanted their impairment or disability to be specifically acknowledged, for example, one participant commented that they wanted society to ‘embrace who I am … and have the right to not feel ashamed or feel different in any way’ (Participant 17). Others wanted to be acknowledged ‘in-spite of’ their impairment or disability and be ‘treated like a human, like I would have been prior to being disabled’ (Participant 3).

Acknowledgement, or lack thereof, occurred on interpersonal (other people and communities) and systemic levels (environments, policies, and system structures) through interactions. Interactions in which acknowledgement occurred were characterised by ‘respect and equal value placed on the unique individual, not only the majority of a given population’ (Participant 6) and resulted in restored dignity – feeling ‘heard and seen as a valuable person’ (Participant 6). Interactions without acknowledgement of personhood resulted in a sense of lost (contingent) dignity.

### Indignity and Lack of Acknowledgement of Personhood

Despite being asked about both dignified and undignified experiences, participants shared many more examples of what was undignified. Participants reported frequent occurrences of undignified interactions in both public and private spaces, but particularly when interacting with the health service system. One participant expressed the frequency with which people with disability are treated without dignity:

Personally, I experience moments that lack dignity daily – specifically by other people or poorly designed places/objects; from multiple disciplines and sources, from people who know and don’t know my journey; health professionals who know or should know/understand diagnoses and symptoms; from people who are able and disabled. Its subtle, often not conscious, not malicious, and sometimes with good intentions. (Participant 13)

The likelihood of undignified experiences was increased in situations in which participants felt particularly vulnerable or dependent, including searching for employment, requiring medical assistance or support, and when another person had authority over information or outcomes. Lack of acknowledgement in these circumstances resulted in participants feeling humiliated, silenced, marginalised, and wholly undignified. One participant described how undignified interactions meant they felt like ‘less of a person, less worthy, less valid, less visible, annoyed, frustrated, sad, angry, no rights, no voice, reducing control’ (Participant 13). Despite the frequency of undignified experiences, participants described practical ways in which dignity could be maintained and enhanced at interpersonal and systemic levels, predominantly through acknowledgement and delivery of human rights as outlined in the articles of the CRPD. These strategies are described in the remainder of the Results section.

### Acknowledgement as the Decision-Maker

Providing opportunities in which participants could express their agency as the decision-maker of their own lives increased the likelihood of maintaining and enhancing dignity. Participants frequently spoke about dignified interactions in which they were able to express their preferences and make independent (although sometimes supported) decisions: ‘We have a story to tell whether we know if it’s true or not. It’s still more beneficial for us to share our point of view’ (Participant 10). Participants commented that dignity was maintained through ‘independence, the right to choose, the right to voice an opinion, a right to live as desired’ (Participant 5), and when they had ‘freedom to live a fulfilling life’ (Participant 12), which might mean having choice and control about how to live life. Even people who required considerable support as a result of their impairment described the need to be acknowledged as the authority or decision-maker of their own lived experience and to voice their preferences without dismissal or erasure: ‘Consider my situation and accept and respect that’ (Participant 1).

Despite the importance of being acknowledged as the decision-maker for dignity, most participants (*n* = 15) reported frequent instances in which interpersonal interactions, particularly with health service staff, featured dismissal, erasure, and silencing of them as the authority of their lives and experiences. For example:

When trying to organise entry to this attraction upon arrival, the person at the gate did not believe that I could be a keyworker and asked to speak to my support worker. In this experience I felt my dignity was eroded. (Participant 8)

Another participant shared that ‘the matre’d chose to speak to my partner/carer about where I would like to eat rather than ask me directly’ (Participant 15). One participant commented that choice and control in relation to the National Disability Insurance Scheme (NDIS) in Australia are ‘just words and [those words are] not being honoured and they’re [NDIS providers] conditioning participants into thinking we have choice and control in scenarios when actually we really don’t’ (Participant 20).

Ten participants reported examples of medical professionals failing to acknowledge their role as a decision-maker by speaking to a support person or family member, rather than directly to them, about their health circumstances, access needs, and support requirements. The impact of lack of acknowledgement often resulted in threats or violations to participants’ dignity. One participant illustrated how lack of acknowledgement as a decision-maker can lead to undignified outcomes. She described a situation during an inpatient hospital stay when:

An allied health person was discussing my condition with another allied health worker and did not include me in the conversation. I was in the meeting but I was referred to as a ‘she’ and was a silent participant. (Participant 1)

The participant went on to say that she felt as though her ‘opinions or comments didn’t matter’ and her mental health and wellbeing was greatly impacted. The pervasive nature of lack of acknowledgement in interpersonal interactions in healthcare contexts can lead to undignified outcomes, especially for people with disability, and can impact negatively on access to quality healthcare.

### Acknowledging My Personhood Through My Right to Access Information

Dignity was maintained and enhanced when participants’ personhood was acknowledged through the delivery of accessible and meaningful information. Provision of complete and transparent information about their medical treatment, care planning, or daily schedule was critical, as was the way in which the information was communicated. Calm, respectful, empathic communication enhanced dignity, as did accessible and inclusive forms of communication, for example, ‘closed captions, SMS, subtitles’ (Participant 17). One participant spoke about the importance of care providers using communication tools that ensured accessible transfer of information: ‘We might need a few tools here and there. Maybe technology to help with communication so we can be educated’ (Participant 07). Another participant stated that access to information may require flexibility and additional considerations to suit the context of healthcare: ‘Find solutions for me to participate as much as I can’ (Participant 01).

All participants mentioned the impact health service staff and other frontline service providers had on dignity, particularly in the ways they shared information and communicated with participants. When help-seeking or negotiating an access challenge, participants highlighted that staff could uphold their dignity by ‘ask[ing] first how best they could help and provid[ing] the help I asked for, rather than doing what they thought I needed’ (Participant 01). Additionally, participants felt their personhood was acknowledged when they were able to ‘respond to a circumstance in an informed way’ (Participant 14). Access to useable information was critical to acknowledging participants personhood and, therefore, enhancing dignity.

### Acknowledging My Personhood Through My Right to Privacy

Participants frequently spoke about maintenance of privacy as an important element of acknowledging their personhood and as a result maintaining their dignity. The right to privacy was typically realised or restricted during interpersonal interactions with health service staff and other service providers, especially when participants were in vulnerable positions, such as relying on staff for assistance, for example, ‘covering naked bodies’ (Participant 3). Eleven participants discussed various ways in which their right to privacy was realised or restricted and the ways in which this impacted their dignity.

Physical and emotional privacy were both important, however, infrequently realised, particularly in hospitals or healthcare systems. Participants recognised that some staff may not have the awareness or education to acknowledge the importance of privacy for people with disability. They also noted that staff were often required to ask participants intrusive questions, albeit with good intentions. However, these questions could require unnecessary disclosures of personal information that increased vulnerability. Participants also reported instances where staff discussed their diagnoses or medical care in public spaces, like hallways. Physical privacy was often transgressed in healthcare environments: ‘When I was younger, a specialist and all his students were there when I was being examined, coming from a shower in a ward naked and wheeled through the ward to my bed as a pre-teen’ (Participant 07).

Although critically important in vulnerable positions in health service contexts, the right to privacy also extended to interpersonal interactions in public and private spaces. Privacy was commonly invaded in interactions in public spaces, outside of health service environments. Three participants mentioned experiences in which their right to privacy was not acknowledged, the most invasive of which was the experience at a beach where ‘people are taking photos [of me]. It is very exposing. People take some appalling images with zero sensitivity’ (Participant 02).

Respecting the right to privacy is a way for people and systems to acknowledge the personhood of people with disability, thereby maintaining and enhancing dignity. Lack of acknowledgement of personhood by not being afforded the right to privacy increased participants' vulnerability and impacted their trust in systems. Most importantly, it reduced their likelihood of returning to hospitals or health environments for follow-up treatment and preventive care.

### Acknowledging My Personhood Through My Right to Accessibility and Inclusion

Participants believed their dignity was maintained when their personhood was acknowledged through efforts to deliver accessibility and inclusion. Eight participants commented that accessibility and inclusion in built and virtual environments enabled them to feel welcome and delivered a sense of belonging, security, and safety. Dignity was maintained when there was an obvious attempt to create a barrier-free physical, attitudinal, and systemic environment.

However, there were few positive examples of participants encountering barrier-free access across mainstream systems and services (transport, education, health, etc.). One participant shared that barriers to access and inclusion were minimised through the use of technology:

I have been introduced to many new technologies and services [in my new role] that I did not know existed. I have been directed to NDIS and Job Access, Microsoft Teams and new hearing aids and technology that will help me in the long term. (Participant 17)

Despite the importance of accessibility and inclusion, the experience of barriers was far more common.

Participants shared that most physical environments and mainstream systems and services were not designed in ways that acknowledged their personhood through accessibility. The impact of inaccessibility and exclusion was profound: ‘[accessibility] is wrong everywhere you look. It’s social exclusion to me. It’s from signage, it’s from walkways. It’s sounds. It’s everywhere you go and shopping centres, everywhere … it’s not dignified at all’ (Participant 21). Daily physical barriers were encountered by most participants, which ‘leads me to feel I don’t belong and I try to find any excuse to leave the environment’ (Participant 22). The lack of equitable physical access was most often described by participants, with one participant being unable to even drop her daughter at school because the education department would not provide wheelchair access. This experience damaged the acknowledgement of her personhood as a mother.

Most participants reported needing a support person, formal or informal, when navigating both physical environments and interacting with systems to increase the likelihood of dignified experiences. The frustration and anxiety generated by inaccessibility cumulated over time. COVID-19 restrictions perpetuated a pervasive culture of indignity, with increased vulnerability, lack of access to services, the use of masks, policies of isolation, and expressions of unworthiness of treatment relative to non-disabled people (see Kendall et al., 2021, for discussion of COVID-19 impacts). Even beyond COVID-19 complications, some participants reported experiencing suicidal ideation in response to the ongoing threats and violations of their dignity through lack of accessibility. These findings highlight the importance of acknowledgement of personhood through efforts to build accessible physical and social environments.

In summary, although dignity can be realised through acknowledgement of a person’s rights to control and choice, privacy, information, accessible environments, and inclusion, the reality experienced by people with disability on a daily basis continues to be one of the pervasive barriers and undignified experiences. Although causality is not clear, participants believe this denial of personhood results in poorer physical and mental health. In contrast, simple acknowledgements of personhood can uphold dignity, encourage people to engage with services and society, and promote positive outcomes.

## Discussion

A global society in which dignity is inherent and experienced equally by all people is not the current reality, despite the human rights imperative to realise this ideal. As described by participants in this study, dignity is defined as the acknowledgement of personhood, which requires the delivery of human rights, particularly in alignment with the rights articulated in the CRPD. Participants described actions through which their personhood could be acknowledged, thus enhancing and protecting their dignity. These actions ranged from positive, empathic interpersonal interactions to the realisation of their human rights at system and service interfaces. Specifically, participants felt their personhood was acknowledged when they were validated, recognised, accepted, and treated with worth and value through acknowledgement of their agency as a decision-maker over their lives; through access to information through which they could make informed decisions; and through welcoming environments and systems that were accessible and inclusive for diverse people. Participants described the strategies that made them feel acknowledged as being direct ‘actionable affirmations’ of their inherent personhood, which is supported in the literature as a critical aspect for dignity (Davis, 2021, p. 2).

While dignity cannot be both inherent and contingent, participants described a bridging of the two concepts, predicated on whether and how the inherent nature of personhood was acknowledged through interpersonal interactions, policies, and the built environment. Participants recognised that dignity is not guaranteed and is assailable, fleeting, and open to threat and violation through lack of acknowledgement of personhood; thus, it is contingent. Personhood, however, is inherent and must be considered as such to underpin human rights. Personhood should be universally recognised ‘by virtue of being born human’ (Arstein-Kerslake, 2021). However, dignity is contingent upon acknowledgement of that personhood. Thus, acknowledgement acts as a connector between the contingent aspect of dignity and the inherent aspects of personhood. Acknowledgement is a complex concept, defined as the acceptance of someone’s existence. Participants described the need to be acknowledged, not in spite of an impairment or identity, not because of it, not for transcending it, but just for being a person, a human being with the same human rights as anyone else.

Acknowledgement of personhood invokes both social and legal connotations, but as described within the Convention on the Rights of Persons with Disabilities is a frame through which people with disability have the imperative for human rights (Arstein-Kerslake, 2021). Personhood is not related to functional or physical capabilities, and impairment should not result in denial of dignity (Pothier & Devlin, 2006; Rioux & Valentine, 2006; Vorhaus, 2015; Yamin, 2016). Acknowledgement of personhood is critically important for people with disability, who have historically been denied basic human rights (Arstein-Kerslake et al., 2021), through erasure of their role as decision-makers with authority over their own lives.

Theoretically, acknowledgement of personhood is often referred to as recognition. Recognition is a well-researched political theory with a range of implications for personhood (Fraser, 2000; Killmister, 2020; Markell, 2003; McNay, 2008; Oliver, 2015; Oliver, 2018; Schick, 2020). Recognition is closely related to respect in the literature; in fact, some theorists combine the two and use terms like ‘recognition respect’ and ‘appraisal respect’ (Allan & Davidson, 2013; Killmister, 2020). Appraisal respect relates to an attitude of positive regard towards other people, which generates some component of moral obligation to consider others during interpersonal interactions (Allan & Davidson, 2013; Killmister, 2020). Although respect was frequently mentioned by participants and sometimes used to describe and define dignity, acknowledgement of personhood appeared to be a broader and more important concept, similar to ‘recognition respect’ (Darwall, 1977; Killmister, 2020). Recognition respect calls for treating people ‘in accordance with [their] membership in a particular social category’ (Killmister, 2020). Participants in this research wanted to be acknowledged and accepted as a whole person, with membership to ‘humanity’. Rather than reflecting a positive respectful interaction, acknowledgement required actions that conveyed the acceptance of the whole person and an understanding of the needs generated by impairment. Indeed, respect was not necessarily required to achieve dignity (see also Hicks, 2021). Acknowledgement is, therefore, more than appraisal, respect, or even moral obligation. It is the embodiment of one’s inherent human rights and personhood and the respect that comes with being recognised as a human being.

Although dignity was contingent on acknowledgement of personhood, it was infrequently experienced. Acknowledgement was sometimes tokenistic or superficial, which felt patronising and belittling. Therefore, it could be potentially problematic for dignity to be contingent on acknowledgement from the systems and people who create and perpetuate the oppressive conditions in which people with disability live (Fraser, 2000; Markell, 2003; McNay, 2008 Oliver, 2015; Oliver, 2018; Schick, 2020). Power is an important element of dignity, in that lack of acknowledgement is more common when power dynamics are unbalanced or uneven (Hicks, 2021; Jacobson, 2009; Schick, 2020). Normative and unbalanced power dynamics are common in healthcare settings, particularly in-patient hospital settings. However, when healthcare practitioners and clinicians acknowledge personhood through the delivery of human rights, the power dynamics become more relational and interdependent rather than dominant and oppressive (Arstein-Kerslake et al., 2021).

## Conclusion

This research has provided some practical and realistic ways in which service providers can address power dynamics and promote acknowledgement of personhood to ensure dignity is maintained and protected. Participants felt most dignified when (1) their personhood was acknowledged; (2) they were acknowledged as the decision-maker in their life; (3) their right to access information was realised; (4) their right to privacy was maintained; and (5) barriers to accessibility and inclusion were removed or minimised. Embedding dignity into practice requires more flexible services and systems. It also depends on co-design and consultation with people with lived experience. However, acknowledgement of personhood is enacted at the interface between people with disability and both service providers and service environments. Without addressing the organisational pressures on staff, lack of resources, and unfounded expectations, opportunities for dignity may still be lost.

There are some limitations to this study. Although all participants focused on the health system as the first and most frequent place in which dignity could be violated, data were not collected to uncover the intricacies of which aspects of the health system were most problematic. Further, the emphasis on the health system meant that undignified experiences in other systems and settings were not explored in detail. Future research should determine the extent to which acknowledgement strategies are applied across different contexts. Finally, there was evidence that dignity was experienced differently by people who had acquired impairment, but this difference requires investigation. It is also important to examine the impact of acknowledgement on wellbeing and quality of life for people with disability. The participants in this study indicated a strong relationship between lack of acknowledgement (and dignity violations) and lower levels of wellbeing. However, the positive impact of acknowledgement and dignity on wellbeing remains untested.
